# Housekeeping genes involved in non-malignant breast phenotypes are widely expressed in multiple cancers and provide novel biomarkers of tumor classification

**DOI:** 10.1590/1414-431X2020e10388

**Published:** 2021-05-17

**Authors:** L. Delmonico, J.C. Obenauer, T.P. Stockfisch, M.V. Fournier

**Affiliations:** 1 Instituto de Biofísica Carlos Chagas Filho, Universidade Federal do Rio de Janeiro, Rio de Janeiro, RJ, Brasil; 2 Rancho BioSciences, San Diego, CA, USA; 3 Bioarray Genetics Inc., Farmington, CT, USA

**Keywords:** Cancer biomarkers, Housekeeping genes, Cancer gene profiling, RNA biomarkers, Oncology biomarkers

## Abstract

Clinically relevant biomarkers are useful to determine cancer patients' prognosis and treatments. To discover new putative biomarkers, we performed *in silico* analysis of a 325-gene panel previously associated with breast epithelial cell biology and clinical outcomes. Sixteen public datasets of microarray samples representing 8 cancer types and a total of 3,663 patients' samples were used for the analyses. Feature selection was used to identify the best subsets of the 325 genes for each classification, and linear discriminant analysis was used to quantify the accuracy of the classifications. A subset of 102 of the 325 genes were found to be housekeeping (HK) genes, and the classifications were repeated using only the 102 HK subset. The 325-gene panel and 102 HK subset were able to distinguish colon, gastric, lung, ovarian, pancreatic, and prostate tumors and leukemia from normal adjacent tissue, and classify disease subtypes of breast and lung cancers and leukemia with 70% or higher accuracy. HK genes have been overlooked as potential biomarkers due to their relative stability. This study describes a set of HK genes as putative biomarkers applicable to multiple cancer types worth following in subsequent validation studies.

## Introduction

Oncogenes and other genes involved in carcinogenesis are well-studied candidates for biomarkers, but some researchers have also looked at models of normal growth, differentiation, or development to identify cancer-relevant genes with some success (1–4). In this context, housekeeping genes (HK) have become a target of research for their relative stability regardless of cell development stages, specific tissue, or external conditions (5,6).

HK are essential genes for basal cell maintenance, regardless of tissue of origin (5). These genes have different evolutionary profiles than others, which contribute to genomic stability. In a previous study by our group, a set of 325 genes was identified as being involved in the formation of organized ductal units in 3-dimensional human mammary epithelial cell culture in laminin-rich extracellular matrix (7). This process includes the transition of cells from a disorganized proliferating state to an organized growth-arrested and polarized state, and these same genes were used successfully to classify breast cancer patients into good and poor prognosis groups (7,8). It has recently been shown that a subset of the 325 gene panel was able to stratify triple negative breast cancer patients into responders to neoadjuvant chemotherapy (NAC), minimal residual disease (RD) after NAC, and even worse-surviving RD cases (9). Moreover, the reproducibility of the 325 RNA biomarkers was validated by comparing two gene expression platforms (Affymetrix and NanoString) (10).

Interestingly, when extending the evaluation of this signature to determine the best method for batch correction of microarray data, it was discovered that a set of 102 genes of the 325 genes are classified as HK (11). A highly cited list of HK genes was defined by the Levanon lab in 2003 (5) and was updated in 2013 (6). In the latter study, the authors identified 3,804 human HK genes, using the criteria that they showed less than four-fold variation in expression across 16 normal human tissues. Then, in this study, the 325 genes were tested for their ability to be used as RNA expression biomarkers in other cancer types besides breast, using either of two criteria: 1) having different expression levels in tumors than in healthy tissue; or 2) having different expression in subtypes of the same cancer. After the initial tests of biomarker capability using the 325 genes, the tests were repeated on the HK subset (102 genes) to determine whether HK genes by themselves can classify tumors.

## Material and Methods

### List of the 325 genes

Samples information from 16 cancer data sets were extracted from the SOFT files for GEO studies (such as GSE26712_family.soft). Sixteen cancer data sets obtained from GEO and ArrayExpress were used to evaluate the expression of the 325 genes (Table 1). For the one ArrayExpress study (E-TABM-157), sample information was extracted from its SDRF file and its associated publication (12). The gene symbols, Entrez Gene IDs, RefSeq accessions, names, and Affymetrix probesets for the 325 genes are shown in Table S1. For genes with multiple Affymetrix probesets, a single probeset was selected to represent the gene. This was typically the probeset showing the highest expression measured in log2 signal intensity.

**Table 1:** Sixteen cancer studies from GEO and ArrayExpress representing 8 cancer types.

Tissue	Accession	N^1^	Scale Factor^2^
Breast (BR1)	GSE25055	310	0.9760
Breast (BR2)	E-TABM-157	51	1.0498
Colon (CO1)	GSE39582	585	0.9660
Colon (CO2)	GSE68468	366	0.8930
Gastric (GA1)	GSE13911	69	1.0006
Gastric (GA2)	GSE54129	132	0.9480
Leukemia (LK1)	GSE13159	568	1.0129
Leukemia (LK2)	GSE14471	110	1.1173
Lung (LU1)	GSE19188	156	0.9731
Lung (LU2)	GSE30219	307	0.9351
Ovarian (OV1)	GSE26712	192	1.0915
Ovarian (OV2)	GSE9891	285	0.9290
Pancreatic (PA1)	GSE15471	78	0.9260
Pancreatic (PA2)	GSE16515	52	0.9926
Prostate (PR1)	GSE17951	154	0.7890
Prostate (PR2)	GSE8218	148	0.8057

Number of samples from each study that were used in this analysis. ^2^Scale factor used for each study in batch correction.

Sample information files were standardized for all 16 studies so that they could be combined conveniently into one large data structure. The individual studies were quantile-normalized using RMA in R and Bioconductor (“oligo” package), and expression levels were converted to the log base 2 scale (13).

The functional analysis of each gene (325) was generated from of the DAVID algorithm (the Database for Annotation, Visualization and Integration Discovery) (14). To describe significant canonical pathways, mutations, evidence of target drug development, biological functions, diseases, and interaction networks, QIAGEN's Ingenuity Pathway Analysis (IPA^®^, QIAGEN, USA) was used. The core analysis was performed and direct and indirect relationships were considered to generate the networks. Canonical pathways were sorted by highest enrichment score and smallest Benjamini-Hochberg-adjusted P-value.

### Cancer data sets

Sixteen public data sets of microarray expression were selected for 8 cancer types: breast, colon, gastric, leukemia, lung, ovarian, pancreatic, and prostate cancers. The data sets were taken from NCBI's GEO and EBI's ArrayExpress resources (15,16). The accession numbers and sample sizes used for each cancer type are shown in Table 1, adding up to 3,563 patients and cell lines. All of the microarrays used were Affymetrix HG-U133A or U133 Plus 2.0. These two platforms share 22,277 probesets in common, and only these common probesets were used in our analyses. The expression range of the 325 genes was examined in all 16 studies. In the 11 studies that included some normal samples (CO1, CO2, GA1, GA2, LK1, LU1, LU2, OV1, PA1, PA2, PR1), the classification of tumor and normal samples was tested. Seven of the studies included cancer subtype information (BR1, BR2, GA1, LK1, LU1, LU2, OV2) and these were used to test subtype classification.

For each classification test, feature selection was used to identify 20-gene subsets of the 325 genes that differed most between the two groups being compared and linear discriminant analysis (LDA) was used to classify the groups (17). Most of the studies are heavily unbalanced, having many more tumor samples than normal ones. In unbalanced cases like these, overall classification accuracy (number of correctly classified samples divided by all samples) is not a useful metric for evaluating model results, because a simple model classifying every sample as a tumor would be 95% accurate when 95% of the samples are tumors. Instead, a class accuracy average was used, where the accuracy of the tumor class was calculated first, then the accuracy of the normal class, and then these two values were averaged.

### Batch correction

The 16 studies were batch corrected in order to compare gene expression levels across cancer types. Correction using ComBat (18) was attempted first, but covariates like disease subtype names were confounded with batches in several cases and ComBat would thus remove subtype variability. Instead, the 16 studies were batch corrected using an equal medians method, constraining the median of the 22,277 probesets to have the same value in each study (11). This preserves the variability of individual genes (like the 325-gene set) while putting the overall expression distributions of the 16 studies on a similar scale. The batch correction scale factor used for each study is included in Table 1.

### Randomized gene sets

To test whether any random set of genes with the same size as the 325 genes would show similar expression profiles, 1,000 random sets of the 325 genes were sampled from the full list of 22,277 probesets. Total expression of each set was calculated as the sum of the average log_2_ intensities of the 325 genes. The gene expression of the 325 genes was also compared to a set of 325 randomly selected HK genes using the same calculations. The random 325 HK genes were selected from only non-325 genes in Levanon's list of 3,804. Table S1 has two tabs, the first showing the 325 genes and the second the 102 HK genes.

### Feature selection

Subsets of the 325 genes that differed the most between tumor and normal conditions were identified by a feature selection method. Two values, TumorHigh and NormalHigh, were computed for each gene as follows: TumorHigh = (T_median_ - N_median_) / (T_SD_ + N_SD_), NormalHigh = (N_median_ - T_median_) / (T_SD_ + N_SD_). T_median_ and N_median_ are the median values of the tumor group and normal group, respectively, and T_SD_ and N_SD_ are their standard deviations. Genes with the top 10 TumorHigh scores were combined with genes having the top 10 NormalHigh scores, and these 20 genes were used as the features in the tumor/normal classification tests. The same feature selection method was used for the disease subtype tests, combining 10 genes higher in one subtype with 10 genes higher in the other subtype.

### Tumor *vs* normal tests

LDA was performed to compare tumor and normal samples for the 11 studies that included normal samples (CO1, CO2, GA1, GA2, LK1, LU1, LU2, OV1, PA1, PA2, and PR1). Samples that were metastases or precancerous biopsies were excluded from the comparisons. Most of the 16 cancer data sets have heavily unbalanced groups being compared, such as 300 cancers compared to only 10 normal samples, which can bias differential expression results (19), and this is why differential expression was not used for our tests. LDA was used with equal prior probabilities, which avoided the bias of unbalanced groups.

### Disease subtype tests

Seven of the studies (BR1, BR2, GA1, LK1, LU1, LU2, and OV2) contained cancer samples with different subtypes. The subtypes tested are listed in Table S2. The same feature selection and LDA methods described above for the tumor *vs* normal tests were used for the subtype tests. In the R scripts for testing tumor/normal differences (tn_diffexp.R) and for testing subtype differences (e.g., ov2_subtypes.R), a maximum of twice as many samples was allowed in one group compared to another. For example, in ovarian cancer, if the input data had 100 serous samples and only 10 endometrioid samples, then 20 of the 100 serous samples were randomly selected to make the comparison 20 of one group *vs* 10 of the other group. This process was automated using R scripts so the rule was applied consistently to all comparisons made in the study.

### Contributions of HK genes

The tumor *vs* normal and disease subtype tests were repeated using the HK subset of the 325 genes (102 genes) to examine whether these genes contributed to the separation of sample groups.

## Results

### Expression of the 325 genes

The expression levels of the 325 genes were plotted for each of the 16 studies. In all studies, the 325 genes were expressed across the full dynamic range of the Affymetrix platform, from log_2_ signal intensity values of about 4 to 12. Using log_2_ <4 as the background threshold, at least 324 of the 325 genes were expressed above background in every cancer type. Figure S1 shows boxplots of the 16 studies before (Figure S1A) and after (Figure S1B) batch correction. Figure S1C and D shows boxplots of the batch-corrected 325 and 102 HK genes.

The minimum, average, and maximum of the 1,000 expression testing results for the 325 genes are plotted for each cancer type in Figure S2. The 325 genes have higher total expression than all 1,000 random sets in every cancer type. The 325 genes also show more variability across cancer types than the random sets do. As another test, 325 other genes were selected at random from Levanon's 3,804 HK genes, where the random selection was constrained to prevent overlap with the 325 genes. The random HK genes were intermediate between the 325 genes and the random gene sets, both in their expression levels and in their variability across cancer types (Figure S2).

### Functional analysis of genes

Functional analysis and grouping by biological function of the 325 genes was performed using DAVID bioinformatics resources (14). From the conversion of the Affymetrix probes to Entrez Format, the algorithm was able to return the function of 320 genes. The functional analysis of the genes is shown in Table S3. Furthermore, 67 gene clusters based on the greatest interactions and biological functions are provided. The clusters with the highest outstanding scores represent overlapping functions, for example, function in the cell cycle, cell division, mitosis, and DNA repair and replication (Table S4).

The analysis of genes using QIAGEN's Ingenuity Pathway Analysis (IPA^®^, QIAGEN) shows the biological function of each gene. The software revealed diseases, expression profiles, molecular changes, and drugs under development that have the genes in question as a target for study (Table S5). In particular, the software has grouped the genes studied here into five major functional groups: cell survival and death (P=5.00E-05), cell cycle (P=6.92E-05), DNA replication, recombination, and repair (P=6.92E-05), cell development (P=7.76E-05), and cell growth and proliferation (P=7.76E-05), reaffirming the data found by the DAVID algorithm previously. The main diseases and disorders related to genes were five: cancer (P=6.92E-05), organismic injury and abnormalities (P=6.92E-05), gastrointestinal disease (P=6.81E-05), reproductive system disease (P=6.92E-05), and endocrine system disorders (P=3.68E-05).

### Tumor *vs* normal tests

The first test was to determine whether the gene panels were able to discriminate normal from tumor tissues. For that, 11 datasets were analyzed that contained both tumor and normal samples. Table 2 shows the tumor/normal classification results for the 325 and 102 genes. The LDA results show that the 325-gene panel correctly classified over 90% of tumor and normal samples in colon, gastric, leukemia, lung, ovarian, and prostate cancers datasets, and 4 of these datasets had higher than 95% accuracy (CO2, GA2, OV1, PR1). Similar results were obtained using the two gastric (GA1, GA2) and pancreatic (PA1, PA2) cancer studies, but more variation is seen between the colon (CO1, CO2) and lung (LU1, LU2) cancer studies. When only the 102 HK genes were used, the accuracy was still 90% or higher in the same datasets, and one pancreatic cancer set (PA2) that was 89% with 325 genes set moved up slightly to 91% with 102 HK set, crossing the 90% threshold. These results showed the 325 and set of 102 HK genes contributed to classifications of normal and tumor tissue in 8 of the 11 datasets analyzed, with accuracy above 90%.

**Table 2:** Overall accuracy (“Overall“), tumor group accuracy (“TumorPct“), normal group accuracy (“NormPct“), and average of the tumor and normal class accuracies (“ClassAvg“) for the 325-gene set and its 102 housekeeping (HK) subset.

Study/Gene Panel	Overall		TumorPct		NormPct		ClassAvg	
	325	HK	325	HK	325	HK	325	HK
CO1	84.23	80.64	84.65	81.33	73.68	63.16	79.17	72.24
CO2	98.4	98.4	98.97	98.97	96.36	96.36	97.67	97.67
GA1	94.2	95.65	92.11	94.74	96.77	96.77	94.44	95.76
GA2	100	100	100	100	100	100	100	100
LK1	91.9	92.78	90.89	91.9	98.65	98.65	94.77	95.28
LU1	94.23	94.23	94.51	93.41	93.85	95.38	94.18	94.4
LU2	80.46	73.94	81.57	75.77	57.14	35.71	69.36	55.74
OV1	100	100	100	100	100	100	100	100
PA1	87.18	83.33	92.31	79.49	82.05	87.18	87.18	83.33
PA2	86.54	90.38	83.33	88.89	93.75	93.75	88.54	91.32
PR1	98.36	95.9	98.18	96.36	100	91.67	99.09	94.02

Red type indicates that the mean accuracy of 325 and 102 genes for LU2 dataset was less than 70%.

### Disease subtype tests

Next, the 325 gene panel and the 102 HK subset were tested for tumor subtype classification ability. For this analysis, 14 comparisons were done using the 7 datasets that included tumor subtype information. Table 3 shows the cancer subtype classification results for the 325 and 102 HK genes. The 325 genes had 90% or better accuracy in distinguishing one pair of leukemia subtypes (AML_NORM and AML_INVT), and three pairs of lung cancer subtypes (squamous *vs* adenocarcinoma in LU1, squamous *vs* large cell in LU1, and squamous *vs* adenocarcinoma in LU2). The 102 HK genes showed reduced ability to distinguish tumor subtypes, reaching only 90.59% for LK1 (AML_NORM *vs* AML_INVT) and only 90.25% for LU1 (squamous *vs* large cell). In the breast cancer results, the comparisons between ER and TRIPNEG (ER positive and triple negative), ER/PR (ER or PR positive), and TRIPNEG in BR1 were very similar as would be expected (88% and 89%, respectively), but the ER and TRIPNEG comparison in BR2 was very different (59%). The BR1 samples came from 310 patients, whereas the BR2 samples are 51 cell lines, which is a likely explanation for the difference. In the lung cancer comparisons, squamous *vs* adenocarcinoma classification was high in both LU1 and LU2, but the subtypes that were only available in LU2 (squamous *vs* small cell, basaloid *vs* carcinoid) were poorly classified. Taken together, the results showed that the 325 panel can provide subtype classifications meeting the 90% threshold in one leukemia dataset and three lung cancer datasets whereas the 102 HK followed the same trend but only met the threshold in half of these datasets.

**Table 3:** Classification results for the 325-gene set and 102 housekeeping (HK) subset.

Study	Subtypes	Overall		Subtype1Pct		Subtype2Pct		ClassAvg	
		325	HK	325	HK	325	HK	325	HK
BR1	ER *vs* TRIPNEG	89.76	84.94	84.09	75	91.8	88.52	87.95	81.76
BR1	ER/PR *vs* TRIPNEG	89.2	88.4	91.41	91.41	86.89	85.25	89.15	88.33
BR2	ER *vs* TRIPNEG	63.89	61.11	45.45	54.55	72	64	58.73	59.27
GA1	MSI *vs* MSS	84.21	68.42	78.95	63.16	89.47	73.68	84.21	68.42
LK1	AML_NORM *vs* AML_INVT	93.4	88.65	93.73	88.32	89.29	92.86	91.51	90.59
LK1	AML_NORM *vs* AML_T1517	65.46	63.92	66.67	65.24	54.05	51.35	60.36	58.3
LK1	AML_NORM *vs* AML_T821	63.94	64.71	65.53	66.95	50	45	57.76	55.98
LK1	AML_NORM *vs* AML_TMLL	59.64	59.9	60.68	61.82	50	42.11	55.34	51.96
LU1	Squamous *vs* Adenocarcinoma	98.61	88.89	96.3	81.48	100	93.33	98.15	87.41
LU1	Squamous *vs* Large Cell	100	91.3	100	96.3	100	84.21	100	90.25
LU2	Basaloid *vs* Carcinoid	92.06	88.89	89.74	92.31	95.83	83.33	92.79	87.82
LU2	Squamous *vs* Adenocarcinoma	52.74	61.64	52.46	63.93	52.94	60	52.7	61.97
LU2	Squamous *vs* Small Cell	69.51	70.73	73.77	75.41	57.14	57.14	65.46	66.28
OV2	Serous *vs* Endometrioid	92.96	90.85	94.7	91.67	70	80	82.35	85.83

The “Subtype” columns list the two subtypes being compared. ER: estrogen receptor positive; ER/PR: estrogen and/or progesterone receptor positive; TRIPNEG: triple negative; MSI: microsatellite instability; MSS: microsatellite stability; AML_NORM: acute myeloid leukemia with normal karyotype; AML_INVT: AML with inv(16)/t(16;16); AML_T1517: AML with t(15;17); AML_T821: AML with t(8;21); AML_TMLL: AML with t(11q23)/MLL. Red type indicates that the mean accuracy of 325 and 102 genes for BR2, GA1, LK1, and LU2 datasets was less than 70%.

## Discussion

HK genes have been widely used for gene expression normalization due to their stable expression regardless of external and pathological conditions (5,6). However, a recent evaluation of the expression of 32 genes classified as HK and applied to 12 different types of cancer has revealed that the *GADPH* gene (traditionally used as HK) showed significant mRNA level alterations in more than half of the cases evaluated (18). These results revealed that HK expression may not follow the stability rule and still vary between tumors. In affirmation of this differential expression, the housekeeping genes of the ribosomal protein undergo variations for prostate cancer cells, as does the β-actin gene for treated colorectal cells (20).

In this context, in the healthy tissues where the 325 genes were originally identified (8), these genes were up-regulated in the disorganized proliferating state and down-regulated in the organized and growth-arrested state. An unexpected result is that of these genes, 102 were HK genes, showing biomarker potential for HK. A second unexpected result was that these differences extended to at least seven other cancer types (colon, gastric, leukemia, lung, ovarian, pancreatic, and prostate). This was consistent with our results showing their expression differences between tumors and normal tissues (see Table 2).

Before this study, it was not recognized that about one third of the 325 genes qualify as HK genes based on their relatively stable expression across tissues. Yet, HK genes were sufficient to separate tumors from normal tissue in seven of the cancer types (colon, gastric, leukemia, lung, ovarian, pancreatic, and prostate), without contributions from non-HK genes. The functions of the HK subset of the 325 genes include mRNA splicing, mRNA export from the nucleus, regulation of response to heat, recruitment of factors to DNA lesions, protein import into the nucleus, and mitochondrial genome maintenance (Tables S3 and S4).

The expression results showed that the 325 genes were expressed at a broad range of levels in 8 different cancer types (breast, colon, gastric, leukemia, lung, ovarian, pancreatic, and prostate) and at higher levels than randomly selected gene lists, despite being originally identified in a breast-specific developmental process. The genes were able to accurately classify tumor and normal samples from colon, gastric, leukemia, lung, ovarian, and prostate cancers, and some subtypes of lung cancer and leukemia. The HK subset was able to classify tumor and normal samples in all of the same cancer types as the 325 set as well as pancreatic cancer, while showing reduced accuracy in subtype classification. These lines of evidence support the potential utility of both the 325 genes and the 102 HK genes as biomarkers in multiple cancer types. Recently, our group showed that the differential expression of 325 genes in breast biopsies in neoadjuvant chemotherapy were able to stratify, surprisingly, the cases with RD at a rate of 83 and 91% in two independent cohorts (519 and 304 cases), comprising different breast tumor subtypes (ER+HER2-, triple negative, HER2+, and ER-HER-PR+). Furthermore, for the triple negative group, a tumor subtype with a worse prognosis and high rates of recurrence, the 325 genes in two different cohorts were able to identify 85.4% (88/103) and 86.2% (56/65) of the cases with RD (9).

This study is original and expands with multiple results from *in silico* analysis. Further work is needed to validate these putative tumor-specific markers for independent experimental and clinical validations, including different tumor subtypes and their molecular variations. The research opens avenues for investigation of other HK genes outside the set of 325 genes that could also classify tumors and normal samples. In addition to tumor/normal classification, further work will focus on specific predictions of interest (diagnosis, prognosis, patient stratification) in order to confirm that the 325 and 102 genes can provide clinical benefits.

## Supplementary Material

Click here to view [pdf].
